# Psychoactive Substance Use and Its Associated Factors among Truck Drivers in Ethiopia

**DOI:** 10.1155/2021/1604245

**Published:** 2021-02-09

**Authors:** Tewodros Yosef, Dawit Getachew, Biruk Bogale, Wondimagegn Wondimu, Nigusie Shifera, Yilkal Negesse, Ameha Zewudie, Wondwossen Niguse, Aragaw Tesfaw, Hadgu Gerensea

**Affiliations:** ^1^Department of Epidemiology and Biostatistics, School of Public Health, College of Medicine and Health Sciences, Mizan-Tepi University, Mizan Teferi, Ethiopia; ^2^Department of Clinical Pharmacy, School of Pharmacy, College of Medicine and Health Sciences, Mizan-Tepi University, Mizan Teferi, Ethiopia; ^3^Department of Nursing, College of Medicine and Health Sciences, Mizan-Tepi University, Mizan Teferi, Ethiopia; ^4^Department of Public Health, College of Medicine Health Science, Debre Tabor University, Debre Tabor, Ethiopia; ^5^School of Nursing, College of Health Science, Axum University, Axum, Ethiopia

## Abstract

**Background:**

Road traffic accidents (RTAs) remain an important public health issue worldwide. Psychoactive substance use is one of the main contributors to the occurrence of traffic accidents, and its use by truck drivers is a global problem. Also, psychoactive substance use is a commonly observed behavior among truck drivers. To the best of our knowledge, no evidence shows the prevalence and factors associated with psychoactive substance use among truck drivers in Ethiopia. Therefore, this study was aimed at assessing the prevalence and factors associated with psychoactive substance use among truck drivers in Ethiopia.

**Methods:**

A cross-sectional study was conducted among 400 systematically selected truck drivers at Modjo dry port in Ethiopia, from February 1 to March 1, 2018. The data were collected through face-to-face individual interviews using a structured questionnaire. The collected data were entered into EpiData version 4.2.0.0 and analyzed using SPSS version 20. Binary logistic regression analysis was computed to determine the association using crude and adjusted odds ratios at 95% confidence intervals. The level of significance was declared at *p* value < 0.05 in the multivariable binary logistic regression analysis.

**Results:**

Of the 400 truck drivers interviewed, the overall one-month self-reported prevalence of psychoactive substance use was 70% (*n* = 280). In the multivariable binary logistic regression analysis, aged 38 years and above (AOR = 0.40, 95% CI [0.23-0.69]), Christianity religion (AOR = 0.52, 95% CI [0.28-0.97]), college and university education (AOR = 3.47, 95% CI [1.27-9.47]), having a family size of 3 or more (AOR = 0.34, 95% CI [0.20-0.60]), having 6 or more hours spent sleeping at night (AOR = 0.46, 95% CI [0.28-0.75]), and rest breaks between driving (AOR = 2.13, 95% CI [1.14-3.97]) were significantly associated with psychoactive substance use.

**Conclusion:**

The one-month prevalence of psychoactive substance use among truck drivers was remarkably high. We can conclude that psychoactive substance use is a public health problem among truck drivers, which is a major threat to themselves and others on the road. The sociodemographic and occupational factors are the factors associated with drivers' psychoactive substance use. Therefore, devising health education and counseling program for drivers to tackle the problem plays paramount importance.

## 1. Introduction

Road traffic accidents (RTAs) remain an important public health issue worldwide. More than 1.2 million people die each year due to road fatalities, and 20 to 50 million are estimated to be injured [1]. Traffic accidents have increased periodically at alarming rates, and it is a serious problem throughout the globe particularly in developing countries like Ethiopia [2]. RTAs were the second most common form of accidents and injuries, accounting for 22.8% of all such incidents next to accidental falls. It contributed to 43.8% of all fatalities secondary to accidents and injuries [3].

Psychoactive substance use is one of the main contributors to the occurrence of traffic accidents [4–7]. Driving under the influence of psychoactive substance use is a major public health concern [8] and has a relevant impact on the drivers' health and safety, increasing the risk of injuries and traffic accidents, potentially affecting the general public health as well [9]. Psychoactive substance use by truck drivers is a global problem [9, 10]. Truck drivers choose psychoactive substances as a form of performance-enhancing drug, to increase productivity [9], to keep awake while driving [11, 12], and to augment their strength with substances as an adjustment strategy to stressful jobs, overcome depression, and overcome daily problems and for pleasure [10, 12].

Drivers who drive under the influence of substances represent a major threat to themselves and others on the road [13–16], since their driving performance is easily impaired as a consequence of the use of substances and has been linked to reckless driving, car crashes, and fatal accidents [17–22].

The factors associated with psychoactive substance use are varied and may include age, marital status, religion, education, income, longer trips, driving in the night shift, long or short sleep duration, fewer hours of rest, little experience of the driver, presence of multiple sex partners, and previous involvement in road traffic accidents [10, 15, 20, 23, 24]. The use of psychoactive substances such as alcohol and chat leaves has become one of the rising major public health and socioeconomic problems worldwide, which is a particularly growing concern in Ethiopia [25]. Psychoactive substances are associated with a multiplicity of noncommunicable diseases and their risks and musculoskeletal disorders [26–28]. Truck drivers are important contributors to the economy of every country, especially in those with limited rail [10], water, and other forms of transport of goods.

Despite this, psychoactive substance use is a commonly observed behavior among truck drivers. To the best of our knowledge, no evidence shows the prevalence and factors associated with psychoactive substance use among truck drivers in Ethiopia. Therefore, this study was aimed at assessing the prevalence and factors associated with psychoactive substance use among truck drivers in Ethiopia to fill the knowledge gap. The findings will help policymakers to devise interventions and also provide opportunities for future studies to fill in the gaps that this study could not address.

## 2. Methods

### 2.1. Study Design, Area, and Period

A cross-sectional study was conducted from February to March 2018 at the Modjo dry port. The Modjo dry port is the first dry port development established at the end of 2009 to relieve the congestion of the Djibouti port. It is found in central Ethiopia, 38 miles southeast of Addis Ababa. The port handles 95% of Ethiopia's trade and is the major bottleneck in the Ethiopia-Djibouti trade corridor [26].

### 2.2. Populations

The source population was all cross-country truck drivers at the Modjo dry port in Ethiopia. The study population was systematically selected truckers at the Modjo dry port in Ethiopia.

### 2.3. Sample Size Determination and Sampling Technique

The minimum required sample size was calculated using a single population proportion formula. Since there was no study regarding psychoactive substance use among truck drivers in Ethiopia, we used an estimated proportion of truck drivers with substance use (50%), precision level 5%, 95% confidence interval, and 10% for nonresponse compensation. The calculated sample size was 422. On average, a maximum of 15 days is required for a truck to make a round trip from the Modjo dry port to Djibouti International Port and back to Modjo dry port unless a technical problem on the vehicle or other accidents occurred. Based on the information from the port management, an average of 300 to 400 trucks arrives daily at the port. With this consideration to give each driver an equal chance of inclusion, the total sample size was divided by fifteen days and concluded that 28 truck drivers can be studied every day. To identify the potential study participants using the systematic random sampling technique, 300 was divided by 28 to obtain the constant for the sampling interval, which was 11. A random number between one and eleven was chosen as a starting number; in this case, it was 6. Hence, every eleventh driver from the 6th driver was studied until the total sample size was obtained. Of the 422 sample size determined, 400 took part in this study, yielding a response rate of 94.8%. The reason for nonresponse (5.2%) was the need for an incentive by respondents; as a result, the previous study by NGO on HIV prevalence had an incentive (5 USD for having HIV testing); some respondents think that the study had no any importance for them and some did not volunteer without any reason.

### 2.4. Study Variables

The dependent variable was psychoactive substance use (at least one of the alcohol/khat). The independent variables were sociodemographic factors (age, marital status, religion, educational status, monthly income, and family size), occupational and psychosocial factors (daily driving hours, years of truck driving, sleeping hours spent at night, rest break between driving, and perceived job stress).

### 2.5. Operational Definitions

- *A psychoactive substance user*: defined as a person who used at least one of the following psychoactive substances such as khat and alcohol to keep awake while driving.
- *Prevalence*: defined as the frequency of study subjects who used psychoactive substances in the past month.
- *Current use*: consuming any substance within the last one month/30 days [29].
- *Lifetime use*: refers to the use of any of the substances at least once in an individual's lifetime [29].
- *A rest break between driving*: defined as a rest taken by a driver after an hour or more of driving but not including rest for the meal.

### 2.6. Data Collection Instrument and Procedures

A face-to-face interview was used to collect the data. First, the questionnaire was prepared in English and then translated to the local language (Amharic) and then translated back into English to maintain its reliabilities. The questionnaire was seen by experts for face validity. Regarding the reliability of the instrument, the questionnaire was tested for a reliability score and a good reliability score was gained (Cronbach's alpha of 0.82 was obtained in this study which resulted in the same). Training was given to data collectors and supervisors regarding the objective and method of data collection and to discuss the presence of unclear questions in the questionnaire.

### 2.7. Data Processing and Analysis

The collected data were entered into EpiData version 4.2.0.0 and analyzed using SPSS version 20 statistical software. Binary logistic regression analysis was computed to determine the association using crude and adjusted odds ratios at 95% confidence intervals. The independent variables with a *p* value of less than 0.25 at the bivariate level were included in the multivariable binary logistic regression model to control for potential confounding. Multicollinearity between predictor variables in the model was checked, and the variance inflation factor (VIF) was found acceptable (less than 2). The Hosmer-Lemeshow goodness-of-fit test indicated (*p* = 0.545) that the model was good enough to fit the data well. The level of significance was declared at *p* value < 0.05 in the multivariable binary logistic regression analysis.

## 3. Results

### 3.1. Sociodemographic Characteristics

Of the 422 truck drivers recruited, 400 truck drivers were successfully interviewed. All respondents were male. Two-thirds (67%) and more than three-fourths (81.2%) of the respondents were married and Christian religious followers, respectively. Two hundred and sixty-seven (66.8%) were secondary (grades 9-12) school achievers. More than half (58.8%) and nearly two-thirds (66.5%) of the respondents were permanent employees and had family sizes of three or more, respectively (Table 1).

### 3.2. Psychosocial and Occupational Factors

Of the 400 truck drivers, nearly three-fourths (75.8%) and two hundred forty-nine (62.2%) had perceived job stress and job satisfaction, respectively. The majority (84.8%) of the respondents had a rest break between driving (Table 2).

### 3.3. Prevalence of Psychoactive Substance Use

Of the 400 truck drivers interviewed, the lifetime prevalence of self-reported prevalence of alcohol drinking and khat chewing was 66% and 34.8%, respectively. The one-month self-reported prevalence of alcohol drinking and khat chewing was 55%, and 30%, respectively. However, the overall self-reported lifetime and one-month prevalence of psychoactive substance use was 83% (*n* = 332) and 70% (*n* = 280), respectively.

### 3.4. Factors Associated with Psychoactive Substance Use

Bivariate analysis was done for exposure variables expected to have an association with the outcome of interest. The exposure variables at a *p* < 0.25 in the bivariate analysis were included in the multivariable binary logistic regression model. The multivariable binary logistic regression model was done, and exposure variables found associated with the outcome of the variable at a *p* value of less than 0.05 were declared significant. In the multivariable binary logistic regression analysis, aged 38 years and above (AOR = 0.40, 95% CI [0.23-0.69]), Christianity religion (AOR = 0.52, 95% CI [0.28-0.97]), college and university education (AOR = 3.47, 95% CI [1.27-9.47]), having a family size of 3 or more (AOR = 0.34, 95% CI [0.20-0.60]), having 6 or more hours spent sleeping at night (AOR = 0.46, 95% CI [0.28-0.75]), and rest breaks between driving (AOR = 2.13, 95% CI [1.14-3.97]) were significantly associated with psychoactive substance use (Table 3).

## 4. Discussion

This study was aimed at assessing the prevalence and factors associated with psychoactive substance use among truck drivers in Ethiopia. The overall prevalence of psychoactive substance use was 70%, 95% CI (65.5%-74.5%). It was higher than 11% [8], 12.7% [22], and 27.6% in Italy [9]. But it was lower than 76% in Kaduna, Nigeria [30]; 81.1% in Kano, Nigeria [31]; and 93.8% in Lokoja, Nigeria [12]. The prevalence of alcohol drinking was 66%, 95% CI (61.4%-70.6%). This finding was in line with 64% in Himachal, India [15]. It was higher than 8.1% [13]; 17.3% in Ilorin, Nigeria [24]; 18.2% in Kaduna, Nigeria [30]; 40% in Punjab, India [15]; and 40.1% among British Colombia drivers [22]. But it was lower than 79.5% in Lokoja, Nigeria [12]. The observed variation in this condition is due to the differences in sociodemographic and lifestyle factors of the study participants across the different studies. The study population may play a great role in the variation observed, since some studies with a low prevalence of psychoactive substance use are associated with short-distance truck drivers compared to this study. Besides, the operational definitions used among researched substances to mean psychoactive substance use are varied across different studies; this study used alcohol and khat to mean psychoactive substance use; however, others used alcohol and other substances to mean psychoactive substance use.

In this study, the age of respondents was significantly associated with psychoactive substance use. Drivers aged 38 years and above were 60% less likely to use psychoactive substances than their counterparts. This finding was in line with previous studies conducted elsewhere [10, 20]. This could be because those who were older are more likely to have a family (wife and child); this makes them more likely to be responsible for their behavior to lead a healthy life with their family by not exposing themselves to unhealthy behavior (psychoactive substance use) that can cause unexpected death due to road traffic accidents. But this was contrary to studies conducted in India and Nigeria [15, 23].

This study also showed a significant association between religion and psychoactive substance use. Being a Christian religious follower was 48% less likely to use psychoactive substances than being Muslim religious followers. This finding was contrary to a study conducted in the Plateau State, Nigeria [23]. Despite this, alcohol consumption was common among Christian drivers. However, the prevalence of khat chewing was high among Muslims. Since use of at least one of the substances (alcohol and khat) categorized an individual as a substance user in this study, the increased prevalence of khat chewing among Muslims results in the high likelihood of Muslim drivers being classified as substance users. This could be the reason for the variation observed between this study and the previous study in Nigeria.

The survey also showed a very strong association between educational status and psychoactive substance use. Truck drivers who had achieved college and university level of education had 3.5 increased odds of using psychoactive substances than their counterparts. This study was in line with studies conducted in Iran and India [15, 20]. This could be explained by the thinking of substance use as a manifestation of modernization, which is mainly observed among higher education graduates as well as students. Of the 280 drivers with psychoactive substance use, 180 (64.3%) had family sizes of 3 or more. Those drivers with family sizes of 3 or more were 66% less likely to use psychoactive substances than drivers with family sizes fewer than 3. This could be because those who had more family members are more likely to take responsibility to restrict themselves from unnecessary substance use to avoid road traffic accidents.

This study also revealed the association between hours of sleep at night and psychoactive substance use. Truck drivers with 6 or more hours of sleep at night were 54% less likely to use psychoactive substances than drivers with fewer than 6 hours of sleep at night. This result was consistent with a study done by Girotto et al. [10]. This could be because those drivers who had fewer than 6 hours of sleep at night are more likely to be depressed and anxious; this makes them take a substance to relieve their psychosocial problems.

Of the 280 truck drivers with psychoactive substance use, 246 (87.9%) drivers had rest breaks between driving. Having a rest break between driving was significantly associated with psychoactive substance use. Truck drivers who had rest breaks between driving were 2.1 times more likely to use psychoactive substances than drivers who had no rest break between driving. This finding was consistent with a study done by Girotto et al. [10]. Drivers may take a rest after a couple of hours of driving after communicating with one another through the phone and deciding where to take a rest. During the rest time, they may take substances to relieve their tension and to relax.

## 5. Conclusion

The one-month prevalence of psychoactive substance use among truck drivers was remarkably high. We can conclude that psychoactive substance use is a public health problem among truck drivers, which is a major threat to themselves and others on the road. The sociodemographic and occupational factors are the factors associated with drivers' psychoactive substance use. Therefore, devising health education and counseling programs for drivers to tackle the problem plays paramount importance.

## Figures and Tables

**Table 1:** Sociodemographic characteristics of the respondents at the Modjo dry port in Ethiopia.

Variables	Categories	Frequency (*n*)	Percent (%)
Age group	<38 years	203	50.8
	≥38 years	197	49.2
Marital status	Out of marriage	132	33.0
	In marriage	268	67.0
Religion	Christianity^∗^	325	81.2
	Muslim	75	18.8
Educational status	Read and write up to primary	92	23.0
	Secondary (grades 9-12)	267	66.8
	College or university	41	10.2
Employment type	Permanent	235	58.8
	Contract	165	41.2
Monthly income	<220 USD	204	51.0
	≥220 USD	196	49.0
Family size	<3	134	33.5
	≥3	266	66.5

^∗^Protestant and orthodox religions. USD: United States dollar.

**Table 2:** Psychosocial and occupational factors of respondents at Modjo dry port in Ethiopia.

Variables	Categories	Frequency (*n*)	Percent (%)
Perceived job stress	Yes	303	75.8
	No	97	24.2
Job satisfaction	Present	249	62.2
	Absent	151	37.8
Rest break between driving	Present	339	84.8
	Absent	61	15.2
Hours spent sleeping at night	Less than 6 hours	196	49
	6 or more hours	204	51

**Table 3:** Bivariate and multivariable analyses of factors associated with psychoactive substance use among respondents at Modjo dry port in Ethiopia.

Variables	Categories	Psychoactive substance use		COR (95% CI)	AOR (95% CI)	*p* value
		Yes	No			
Age group	<38 years	155	48	1	1	
	≥38 years	125	72	0.54 (0.32-0.78)^∗∗^	0.40 (0.23-0.69)	0.001
Religion	Muslim	58	17	1	1	
	Christian	222	103	0.63 (0.35-1.14)^∗^	0.52 (0.28-0.97)	0.039
Educational status	Read & write up to G-8	58	34	1	1	
	Secondary (grades 9-12)	188	79	1.40 (0.85-2.30)^∗^	1.21 (0.69-2.10)	0.509
	College & university	34	7	2.85 (1.14-7.12)^∗∗^	3.47 (1.27-9.47)	0.015
Family size	<3	100	34	1	1	
	≥3	180	86	0.71 (0.45-1.09)^∗^	0.34 (0.20-0.60)	<0.001
Monthly income	<220 USD	149	55	1	1	
	≥220 USD	131	65	0.74 (0.48-1.14)^∗^	0.86 (0.52-1.41)	0.544
Hours spent sleeping at night	Less than 6 hours	150	46	1	1	
	6 hours or more	130	74	0.54 (0.33-0.78)^∗∗^	0.46 (0.28-0.75)	0.002
Rest break between driving	Yes	246	93	2.10 (1.20-3.67)^∗∗^	2.13 (1.14-3.97)	0.018
	No	34	27	1	1	
Perceived job stress	Yes	207	96	0.71 (0.42-1.19)^∗^	0.84 (0.48-1.48)	0.556
	No	73	24	1	1	

AOR: adjusted odds ratio; CI: confidence interval; COR: crude odds ratio; G-8: grade 8; USD: United States dollar. ^∗^Significant at a ^∗^*p* value < 0.25; ^∗∗^significant at a ^∗^*p* value < 0.05.

## Data Availability

The datasets used and/or analyzed during the current study are available from the corresponding author upon reasonable request.

## Abbreviations

AOR: Adjusted odds ratio
CI: Confidence interval
COR: Crude odds ratio
SPSS: Statistical Package for the Social Sciences
SD: Standard deviation.
